# The causal relationship between immune cells and hepatocellular carcinoma: a Mendelian randomization (MR)

**DOI:** 10.3332/ecancer.2024.1794

**Published:** 2024-11-08

**Authors:** Pengkhun Nov, Yangfeng Zhang, Duanyu Wang, Syphanna Sou, Socheat Touch, Samnang Kouy, Virak Vicheth, Lilin Li, Xiang Liu, Changqian Wang, Peizan Ni, Qianzi Kou, Ying Li, Chongyang Zheng, Arzoo Prasai, Wen Fu, Wandan Li, Kunpeng Du, Jiqiang Li

**Affiliations:** 1 Department of Radiation Oncology, Oncology Center, Zhujiang Hospital of Southern Medical University, No 253 Mid Gongye Ave, Haizhu District, Guangzhou 510282, Guangdong Province, China; 2 Department of Oncology, The People's Hospital of Hezhou, No. 150 Xiyue Street, Babu District, Hezhou City 542800, Guangxi, China; 3 Department of Medical Oncology, The People's Hospital of Hezhou, No. 150 Xiyue Street, Babu District, Hezhou City 542800, Guangxi, China; a https://orcid.org/0000-0002-0684-7291; b https://orcid.org/0000-0002-585-5911; † These authors contributed equally to this work

**Keywords:** hepatocellular carcinoma, immune cells, MR, GWAS study

## Abstract

**Objective:**

Hepatocellular carcinoma (HCC) is a complex and multifaceted disease that is increasingly prevalent globally. The involvement of immune cells in the tumour microenvironment has been linked to the progression of HCC, but the exact cause-and-effect relationship is not yet clear. In this study, we utilise Mendelian randomization (MR) to investigate the potential causal links between immune factors and the development of HCC.

**Method:**

We executed a comprehensive MR study, leveraging publicly accessible genetic datasets to explore the potential causal links between 731 types of immune cells and HCC. Our analysis primarily applied inverse variance weighting and weighted median methods. To evaluate the robustness of our findings and probe for the presence of heterogeneity and pleiotropy, we also conducted thorough sensitivity analyses.

**Results:**

We found 36 immune cells were associated with HCC, CD64 on CD14− CD16+ monocytes (OR = 1.328, 95% CI = 1.116− 1.581, *p* = 0.001), CD3− lymphocyte %lymphocytes (OR = 1.341, 95% CI = 1.027− 1.750, p = 0.031), HLA DR on CD14+ monocytes (OR = 1.256, 95% CI = 1.089− 1.448, *p* = 0.002), CD19 on CD19 on Plasma Blast−Plasma Cell (OR = 1.224, 95% CI = 1.073− 1.396, *p* = 0.003), CCR2 on monocytes (OR = 1.204, 95% CI = 1.073− 1.351, *p* = 0.002) and Naive CD4+ T cell Absolute Count (OR = 0.797, 95% CI = 0.655− 0.969, *p* = 0.023) were the most strongly associated with HCC. Among them, CD64 on CD14− CD16+ monocytes, CD3 − lymphocyte %lymphocytes, HLA DR on CD14+ monocytes and CD19 on Plasma Blast−Plasma Cells are the risk factors, while Naive CD4+ T cell Absolute Count are protective factors for HCC.

**Conclusion:**

Our MR analysis of the role of immune cells and HCC provides a framework for knowledge of circulating immune status. Systematic assays of infiltrating immune cells in HCC can help dissect the immune status of HCC, assess the current use of checkpoint blockers, and most importantly, aid in the development of innovative immunotherapies. Further research is necessary to validate these findings and explore the underlying mechanisms that influence the immune response to HCC.

## Introduction

Hepatocellular carcinoma (HCC) is the most common type of primary liver cancer and typically develops against a backdrop of chronic liver diseases stemming from infections with Hepatitis B (HBV) or Hepatitis C virus (HCV), excessive alcohol consumption or non-alcoholic steatohepatitis (NASH) [1]. Particularly in Western nations, NASH is becoming the leading cause of HCC, linked to metabolic syndrome and diabetes, and this trend underscores the classification of HCC as an inflammation-driven cancer [2]. With nearly a million new cases globally [3] and an estimated 30,000 deaths in the United States for the year 2023 alone [4], liver cancer represents a significant health issue. Treating HCC is a complicated matter due to the cancer’s variability and its frequent occurrence alongside other liver diseases [1]. One distinctive aspect of liver cancers is that they can often be diagnosed without a biopsy, an uncommon practice for solid tumours. Moreover, liver transplantation is considered a mainstay treatment option, especially because over 80% of HCC patients suffer from concurrent liver disease or dysfunction [1]. While therapies such as sorafenib and regorafenib have been shown to offer limited enhancements in survival [5, 6], the overall effectiveness of anti-cancer treatments in HCC remains suboptimal. Even though immunotherapies have shown significant benefits in treating various cancers, their success rate in HCC patients is notably lower [7]. Given that the efficacy of treatments can be linked to the immune landscape within the tumour [8], it is crucial to analyse the immune environment present in HCC. This involves examining the nature and makeup of immune cells within the tumour as opposed to those in other parts of the body where immune activity is relevant.

While numerous cross-sectional and cohort studies have investigated the relationship between immune cells and HCC cancer, their observational nature limits them to establishing correlations rather than causations [9–11]. Although randomised controlled trials (RCTs) could infer causation, interventions to manipulate immune cells are neither feasible nor ethical, thus constraining our ability to draw causal inferences. Considering the scarcity of data from observational and interventional studies, the application of Mendelian randomization (MR) within the field of human genetics provides a valuable avenue for rigorously investigating the potential causal relationships between elevated levels of immune cells and HCC [12]. This approach leverages the random allocation of genetic variation at conception, well before the onset of disease, making MR a valuable tool for establishing causality and mitigating the risk of reverse causality, independent of confounders typically present in study designs [13].

Due to biases in traditional observational epidemiological research designs, the association results obtained from observational studies are susceptible to confounding factors (such as gender and age) and reverse causality (such as lifestyle changes due to HCC), resulting in unreliable causal inference results [14]. MR has been a popular genetic epidemiological research method in recent years. It introduces the concept of instrumental variables (IVs) and uses genetic variations such as single nucleotide polymorphisms (SNPs) as IVs for studying exposure factors, to make causal inferences about given exposure and outcomes. Due to the random allocation of alleles to offspring, confounding factors will not affect the causal association estimates obtained from MR studies; Since genes are determined before birth, diseases cannot affect genes, so MR research can effectively control the biological effects of reverse causality [15].

MR analysis is a technique that leverages genetic variants as IVs to assess the causal relationships between environmental factors and outcomes. By assuming that genetic variants are randomly inherited and not influenced by diseases, this approach effectively mitigates the impact of confounding factors and reverse causation bias [15]. Our analysis was informed by up-to-date statistical summaries derived from a genome-wide association study (GWAS) that concentrated on immune cell traits [16]. Our study is focused on investigating the potential causal links between 731 types of immune cells and HCC, particularly examining their roles in tumour onset, advancement and resistance to treatment. We present an extensive MR study that not only identifies specific immune cells associated with HCC cancer but also addresses the constraints in current research. Our goal is to provide valuable insights that could refine future immune cell methodologies and advance etiological research. This study is intended to support precision prevention, control and the development of innovative therapeutic approaches.

## Materials and methods

### Study design

**Figure 1.**
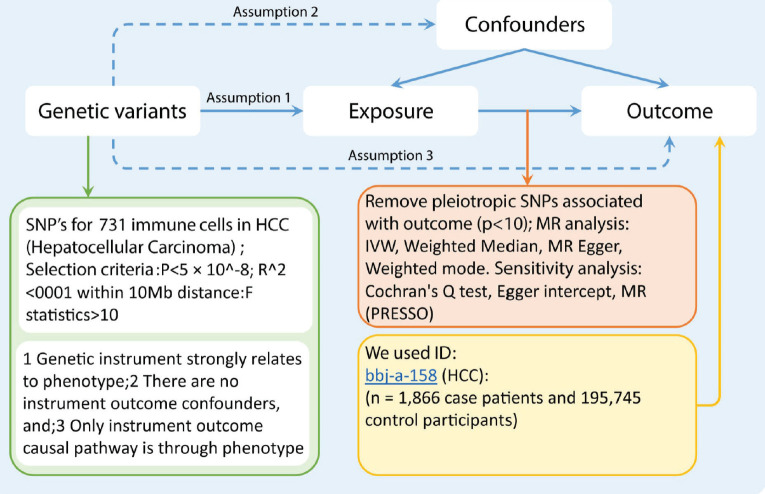
The flowchart of study design. The first assumption is that the instrument variables are strongly related to the exposure. The second assumption specifies that the instrument variables are not associated with any confounders. The third assumption establishes that the instrument variables influence the outcome solely through the exposure. Abbreviations include SNPs for single-nucleotide polymorphisms, LD for linkage disequilibrium, IVW for inverse variance weighted, and weighted median, MR-Egger and MR-PRESSO.

In our investigation, we employed a two-sample MR approach to assess the potential causal effect of 731 immune cell types on HCC. MR leverages genetic variants as IVs to proxy for modifiable risk factors. For MR to provide a valid causal inference, t7he chosen IVs must meet three critical criteria: (1) The genetic variants must have a strong association with the risk factor of interest; (2) The variants should be independent of confounders that could affect both the risk factor and the outcome; (3) The influence of the genetic variants on the outcome must operate solely through the risk factor, without any alternative causal pathways Figure 1.

### Data sources for exposure and outcome

A summary of GWAS statistics for each immune trait is publicly accessible from the GWAS catalog (accession numbers: GCST0001391 to GCST0002121) [19]. We used cancer’s keywords to find the immune traits from (https://gwas.mrcieu.ac.uk/). The immune traits included: bbj-a-158 (HCC). A GWAS is a research approach used to identify genetic variants associated with specific diseases or traits. Unlike earlier methods that focused on single genes or small groups of genes, GWAS examines a large number of genetic variants across the entire genome of many individuals to find associations with particular traits or conditions. GWASs detect common genetic variants that are associated with complex disorders, with the ultimate goal of developing translational prevention or treatment strategies. With their comprehensive coverage of common SNPs and comparatively low cost, GWAS is an attractive tool in clinical and commercial genetic testing. In summary, GWAS is a powerful tool that has significantly advanced our understanding of the genetic basis of complex traits and diseases, providing a foundation for future research and medical advancements. Based on the ID of cancer, we used online data from GWAS including 197,611 Japanese individuals (*n* = 1,866 patients and 195,745 control participants) for HCC to analyse the relationship between immune cells and HCC according to IDs (https://www.ebi.ac.uk/gwas/).

### Instrument selection

In response to the substantial number of SNPs achieving genome-wide significance (*p* < 5 × 10^-8) for immune cell traits, we implemented more stringent criteria (*p* < 5 × 10^-9) for the selection of genetic IVs [16]. These IVs were identified through clustering based on the Linkage Disequilibrium (LD) reference panel from the 1,000 Genomes Project, employing an LD threshold of *R*^2 < 0.001 within a 10,000 kilobase (kb) window. Given the relatively limited scale of GWASs data for immune cells, we adopted a more relaxed *p*-value threshold of 1 × 10^-5 along with a corresponding LD clustering threshold (*R*^2 < 0.001 across 10,000 kb) [17]. To ensure the robustness of our instruments, IVs with *F*-statistics exceeding ten were selected, indicating their suitability for further analysis. These IVs were subsequently extracted from the summary data on HCC outcomes, excluding any IVs demonstrating potential pleiotropic effects on HCC (*p* < 10^-5), in accordance with methodologies from previous studies [18]. For analytical consistency, we harmonised SNPs between the exposure and outcome datasets to ensure concordant effect estimations for the same alleles. SNPs with intermediate effect allele frequencies > 0.42 or those incompatible with the allele in question were excluded from our analysis [17].

### Statistical analysis

In our study, we utilised a variety of genetic variants as IVs, rather than relying solely on an allele score. We chose this approach to thoroughly investigate key assumptions, uncover potential pleiotropy and enable more robust sensitivity and multivariable MR analyses [13]. To evaluate the consistency of our findings under different assumptions regarding heterogeneity and pleiotropy, we employed four distinct MR methodologies: the IVW method using a random-effects model, the weighted median approach, MR-Egger and MR pleiotropy residual sum and outlier (MR-PRESSO). The IVW method, based on a random-effects model, was the primary analytical framework for all four sets of IVs. We assessed heterogeneity using Cochran’s *Q* statistic.

Additionally, our study encompassed analyses with more stringent criteria. The IVW method, assuming the validity of all genetic variants, may introduce bias if numerous SNPs are affected by horizontal pleiotropy [19]. In contrast, the weighted median approach, which is effective when fewer than 50% of variants display horizontal pleiotropy, assumes the validity of the majority of genetic variants [20]. In instances where over 50% of variants are influenced by horizontal pleiotropy, we assessed the strength of our genetic instruments using *F* statistics, with a mean *F*-statistic of less than 10 indicating weak IVs [21].

Moreover, we utilised the MR-Egger method to examine potential directional pleiotropy. A significant intercept in this method would indicate a violation of IV assumptions, suggesting the presence of directional pleiotropy [22]. We also employed the PRESSO method, which is designed to minimise heterogeneity in causal effect estimates by excluding disproportionately influential SNPs (with NbDistribution = 1,500) [23]. Additionally, we conducted Steiger-filtering analyses to identify and remove genetic variants that were more strongly associated with the outcome than the exposure, indicating potential reverse causality [24].

All statistical analyses were conducted using R version 4.3.1 (R Foundation) and specific R packages (‘TwoSampleMR’ and ‘MR’) tailored for MR analysis [25, 26].

## Results

**Figure 2.**
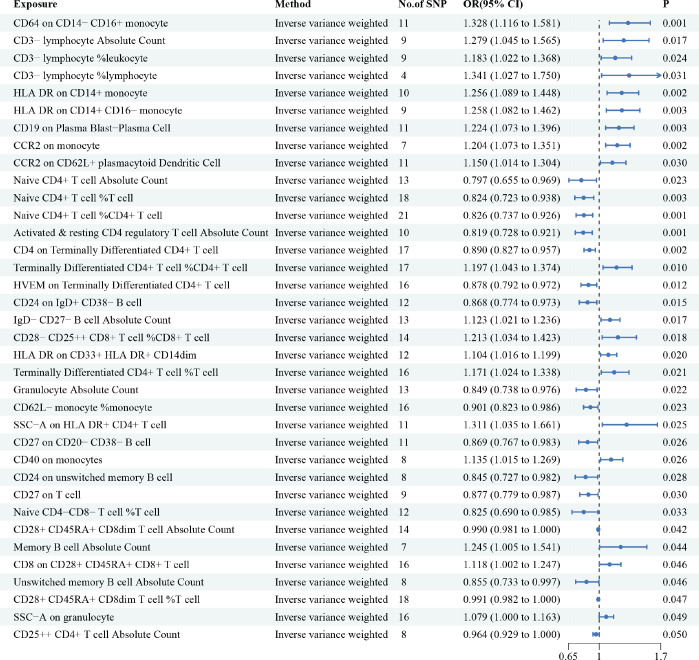
The causal relationship between immune cells and HCC. We selected IVW as a primary method *p* < 0.05 showed statistically significant; OR value >1 indicated a risk factor; OR value <1 indicated a protective factor.

Figure 2 summarises the estimated causal effect of immune cells on HCC susceptibility and utilised IVW as the primary analysis method. Our results showed that 36 immune cells were associated with HCC. We have highlighted some of the strongest associations in the following include CD64 on CD14− CD16+ monocytes (OR = 1.328, 95% CI = 1.116− 1.581, *p* = 0.001), CD3− lymphocyte %lymphocytes (OR = 1.341, 95% CI = 1.027− 1.750, *p* = 0.031), HLA DR on CD14+ monocytes (OR = 1.256, 95% CI = 1.089− 1.448, *p* = 0.002), CD19 on CD19 on Plasma Blast−Plasma Cell (OR = 1.224, 95% CI = 1.073− 1.396, *p* = 0.003), C-C motif chemokine receptor 2 (CCR2) on monocytes (OR = 1.204, 95% CI = 1.073− 1.351, *p* = 0.002), Naive CD4+ T cell Absolute Count (OR = 0.797, 95% CI = 0.655− 0.969, *p* = 0.023). Although heterogeneity was noted, as Cochran’s *Q* yielded a* p*-value of less than 0.05, the causality estimates were satisfactory when using a random-effects IVW approach. *p*-values for the MR-Egger intercept were above 0.05, suggesting that there were no significant pleiotropy effects (Supplementary Tables 1 and 2).

**Figure 3.**
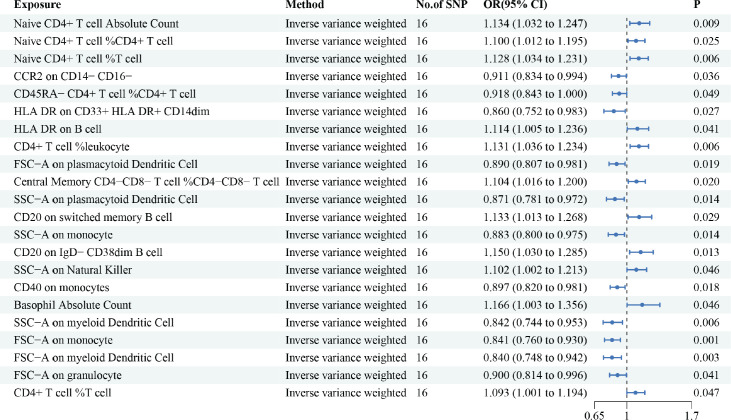
The reverse causal relationship between immune cells and HCC. We selected IVW as a primary method *p* < 0.05 showed statistically significant; OR value >1 indicated a risk factor; OR value <1 indicated a protective factor.

However, in the reverse MR analyses, we found that 22 immune cells were correlated with HCC. Briefly, we have highlighted some of the strongest relations with the HCC risk include Naive CD4+ T cell Absolute Count (OR = 1.134, 95% CI = 1.032– 1.247, *p* = 0.009), CD40 on monocytes (OR = 0.897, 95% CI = 0.820– 0.981, *p* = 0.018) and HLA DR on CD33+ HLA DR+ CD14dim (OR = 0.860, 95% CI = 0.752– 0.983, *p* = 0.027) (Figure 3). Moreover, this article provides a summary of the sensitivity analysis results. Despite observing heterogeneity and obtaining a *p*-value of less than 0.05 with Cochran’s *Q* test, the causality estimates were deemed acceptable when employing a random-effects IVW approach. Additionally, the *p*-values for the MR-Egger intercept exceeded 0.05, indicating the absence of significant pleiotropy effects (Supplementary Tables 3 and 4).

## Discussion

MR analysis has been frequently employed to illustrate possible causality between risk factors and diseases. In the present study, we used MR to generate proof of an inverse causal relationship between immune cells and HCC. In our study, we found a total of 36 immune cells associated with HCC. Among 36 immune cells associated with HCC, CD64 on CD14− CD16+ monocyte, CD3− lymphocyte Absolute Count, CD3− lymphocyte %leukocyte, CD3− lymphocyte %lymphocyte, HLA DR on CD14+ monocyte, HLA DR on CD14+ CD16− monocyte, CD19 on Plasma Blast−Plasma Cell, CCR2 on monocyte, CCR2 on CD62L+ plasmacytoid Dendritic Cell, Terminally Differentiated CD4+ T cell %CD4+ T cell, IgD− CD27− B cell Absolute Count, CD28− CD25++ CD8+ T cell %CD8+ T cell, HLA DR on CD33+ HLA DR+ CD14dim, Terminally Differentiated CD4+ T cell %T cell, side scatter area (SSC−A) on HLA DR+ CD4+ T cell, CD40 on monocytes, Memory B cell Absolute Count, CD8 on CD28+ CD45RA+ CD8+ T cell, SSC−A on granulocyte are risk factors for HCC, while the rest of immune cells are protective factors for HCC. We have highlighted some of the strongest associations in the following discussion sections include CD64 on CD14− CD16+ monocytes, CD3− lymphocyte %lymphocytes, HLA DR on CD14+ monocytes, CD19 on Plasma Blast−Plasma Cell, CCR2 on monocytes, Naive CD4+ T cell Absolute Count.

CD64 (FcgRI) is a Fc IgG receptor that is continuously exposed to macrophages and monocytes. It is a high-affinity receptor for single IgG or Ig in immunocomplexes, and it induces immune and inflammatory responses in immunocompetent cells (i.e., monocytes and syncytial macrophages) [27–29]. In the human body, monocytes constitute a diverse population of cells with three different subpopulations that are based on CD14 and CD16 expression levels [30]. The CD14++ CD16- classical subdivision is the most common of all the circulating monocytes, while CD14- CD16+ cells have usually been characterised as non-classical or mesenchymal monocytes. They are a subpopulation of monocytes that are distinct from classical monocytes and that have distinct functional characteristics. A second subpopulation of monocytes simultaneously expresses both CD14 and CD16 (CD14+ CD16++) and are referred to as intermediate monocytes. The third subpopulation consists of the atypical monocytes, which exhibit low CD14 expression and high CD16 (CD14+ CD16++) expression [31]. In rheumatoid arthritis (RA), CD64 is evaluated by overexpression of CD14++CD16- and CD14++CD16+ monocytes and correlates with the risk of disease activation [32]. CD64 has also been shown to be elevated in chronic HBV-infected individuals and to vary with disease duration. Treatment with interferon-α corrected these elevations, however, suggesting that CD64 levels could serve as biomarkers for both chronic HBV infection and the efficacy of interferon-α therapy [33]. As a member of the immunoglobulin superfamily, CD64 has the high affinity FC receptor type I of IgG, which can mediate cell phagocytosis, antigen presentation, antibody-dependent cell-mediated cytotoxicity, cytokine release and peroxide formation by binding with IgG, and thus participate in the body’s immune response [34]. Studies have shown that CD64 protein has tissue-targeting peptide and humanised monoclonal antibody, and the combination of CD64 protein-containing extracellular vesicles (dtEV) and low-dose chemotherapy drug gemcitabine can effectively inhibit the growth and metastasis of pancreatic ductal adenocarcinoma in mice, and prolong the survival rate of the animals [34]. Confirmation of communication between CD64-loaded extracellular vesicles (dtEV) associated cells suggests that CD64 is one of the potential anti-tumour potential targets. Our findings indicated that CD64 served as a risk factor in HCC.

CD3− lymphocyte %lymphocyte indicates a measurement or quantification of a subset of lymphocytes. Specifically, it refers to lymphocytes that do not express the CD3 molecule, which is commonly found on the surface of T cells [35]. Recent studies have demonstrated that the percentage frequency of CD3+ T cells in tumour samples is correlated with complete response and partial response, and that an increase in CD3+ cells is associated with improved overall survival. Previous gene expression profiling data have demonstrated the significance of T-cell depletion and tumour T-cell infiltration in HCC and have shown that CD3+ T-cell depletion correlates with responses to nivolumab [36]. In colorectal cancer (CRC), Galon *et al* [37, 38] showed that a greater concentration of CD3-positive immune cells was associated with improved survival. High granzyme B and CD4RO-positive immune cell concentrations were also associated with improved survival rates. CD3 also had the greatest predictive potential in their study when compared to other biomarkers. Another study showed that low CD3 cell density was associated with a worse prognosis, but it was still an equivalently potent prognostic marker [39]. Moreover, high CD3-positive inflamed cells have been associated with improved survival in CRC [40]. In immature T lymphocytes, CD3 / T-cell receptor (TCR) recognises endogenous antigen peptides presented by the major histocompatibility complex to trigger signals that enable immature T lymphocytes to undergo positive and negative selection in the thymus. They become peripheral naive T lymphocytes. In mature T lymphocytes, foreign antigen peptides bind to the MHC to activate the CD3 /TCR complex, which is a critical step in the differentiation of antigen-specific T lymphocytes into effector or memory T lymphocytes. In this process, CD3ε plays a key role, and blocking CD3ε causes T lymphocytes to stay in the double-negative phase. CD3 targets participate in many signaling pathways, mainly the following: TCR signaling pathway: TCR recognises and binds to antigenic peptides presented by MHC molecules, resulting in tyrosine residues of the ITAM conserved sequence of CD3 being phosphorylated by tyrosine protein kinases in T cells. Lymphocyte-specific protein tyrosine kinase (Lck) via CD3ε recruitment controls the initiation of TCR phosphorylation, thereby regulating cytokine production, cell survival, proliferation and differentiation [41–43]. PI3K-Akt signaling pathway: After CD3 target activation, it can promote the proliferation and survival of T cells and regulate the immune response of T cells by activating the PI3K-Akt signaling pathway [44–46]. NF-κB signaling pathway [47]: After CD3 target activation, it can promote the proliferation, differentiation and survival of T cells by activating the NF-κB signaling pathway, and regulate the immune response of T cells; MAPK signaling pathway: After the CD3 target is activated, the MAPK signaling pathway can be activated to promote the proliferation, differentiation and functional regulation of T cells [48, 49]. Ca2+ signaling pathway: After activation of the CD3 target, it can promote the proliferation, differentiation and functional regulation of T cells by activating Ca2+ signaling pathway, and regulate the immune response of T cells. Our findings are consistent with the conclusion that CD3− lymphocytes are a risk factor for HCC and may promote the development of HCC [50]. Our present study also showed that CD3− was a risk factor for HCC, but the mechanism research and functional research are need to further validated in the future research.

HLA DR on CD14+ monocyte represents an analysis or measurement performed using flow cytometry to assess a specific characteristic of monocytes with CD14 expression in relation to the scatter parameter SSC-A. CD14 serves as a pattern recognition receptor that enhances immune reactions within the innate immune system. Initially identified as a marker for monocytes, CD14 triggers cellular responses when it comes into contact with bacterial elements. Due to the lack of an endocytic tail, CD14 was suspected to be signaling capable. CD14 was subsequently demonstrated to be a Toll-like-receptors (TLRs) co-receptor that functioned to assay pathogen-related molecular patterns. CD14 is now understood to be a versatile receptor, however, as it has recently been found to activate the nuclear factor of activated T cells (NFAT), modulate myeloid cell life cycle in a TLR4-dependent manner, and transit inflamed lipids and induce phagocyte hyper-activation. The effects of CD14 on a variety of related diseases have also been investigated [51]. CD14 has been shown to be related to tumour relapse, proliferation, metastasis and chemoresistance, all of which are features of cancer stem cells. Therefore, it was hypothesised that esophageal hematopoietic stem cells (EC) also display CD14. In a recent study, human EC sections were paraffin-embedded and the co-expression of CD14 and then analysed for the concurrent expression of CD14 and EC marker aldehyde dehydrogenase 1 (ALDH1) through the use of immunofluorescence staining. CD14+ cells were then separated using immunomagnetic separation for stemness assays, which included proliferation, migration, invasion and tumourigenicity. Proliferative abilities were detected using the Cell Counting Kit 8, EdU and colony formation assays, metastatic abilities were detected using Transwell and wound healing assays, and tumourigenic abilities were detected with xenograft assays. The results showed that ALDH1-labeled EC exhibited CD14 and that primary CD14+ cells were characterised by CSCs. Thus, this study suggested that CD14 could also serve as a cellular surface marker for EC [52]. Our findings indicated that the presence of HLA DR on CD14+ monocyte was a risk factor in HCC. More functional research is needed to confirm these findings.

CD19 on Plasma Blast−Plasma Cell is a cell surface protein that is commonly used to identify B cells, which are leukocytes that perform important immune system functions (e.g., producing antibodies). CD19 expression is triggered at the onset of the B lineage during hematopoietic stem cell derivation and persists through the derivation of precursor and mature B cells until it is downregulated at the time of syngeneic plasma cell derivation [53]. Several studies have reported that elevated levels of CD19+ cells may serve as a useful reference value for surveillance of immunologic function in some populations at risk for HCC. Decreases in the number of CD19+ cells in HCC patients suggest a decrease in the body’s capability to fight viruses, which can eventually lead to disease progression because of sustained viral infection [54]. As an important marker, CD19 is widely used in the diagnosis and prognosis of leukemia, lymphoma and immune system diseases [55–58]. At present, the main clinical means to treat malignant tumours with CD19 as the target are chimeric antigen receptor T cell (CAR-T) therapy and antibody-coupled drugs [59, 60]. It was found that N125 low glycosylation of CD19 would lead to the loss of protein expression, which seriously impaired the efficacy of CAR-T cells against leukemia, and N125 high glycosylation of CD19 would prevent the activity of CAR-T, thus making CAR-T treatment resistant. Blocking the hyperglycosylation of N125 enhances binding to the FMC63 antibody [61]. In addition, studies have shown that CD19+ macrophage subpopulation exists in liver cancer, which enhances mitochondrial metabolism by upregulating the expression of transcription factor PAX5 [62], thereby inhibiting lysosome function, increasing the expression of immunosuppressive proteins CD73 and PD-L1 on cell surface, inducing the formation of immunosuppressive microenvironment and promoting the progression of liver cancer, confirming that cell therapy targeting CD19+ macrophages can significantly improve the immunotherapy effect of liver cancer. Our study also showed that CD19 expression is strongly associated with HCC risk.

CCR2 is predominantly expressed on the surface of monocytes/macrophages and lymphocytes. It acts as a regulatory receptor for functional chemotaxis and plays a role in the modulation of various diseases by controlling the migration of bone marrow monocytes into the bloodstream and their subsequent movement to sites of inflammation [63, 64]. In the context of the liver, CCR2 is implicated in several hepatic pathogenic processes, including acute liver injury, chronic hepatitis, liver fibrosis/cirrhosis and tumour progression [65–67]. CCR2 can contribute to hepatic fibrosis by influencing the mobilization of circulating monocytes to damaged hepatic cells and hepatic stellate cells (HSCs) through the activation of HSC [66, 67]. Furthermore, CCR2 serves as a high-affinity receptor for members of the monocyte chemotactic protein family, such as C-C motif chemokine ligand 2 (CCL2), CCL7, CCL8, CCL12 (in mice only) and CCL13 (in humans only) [68–72]. Given its crucial role in hepatic homeostasis and disease, research over the past decade has heavily focused on modulating CCR2 expression. Various upstream regulators include inflammatory cytokines like TNF, IL-6, IL-1β and IFN-γ [73]. CCL2/CCR2 is a key pathway that regulates the recruitment of macrophages from monocytes in solid tumours. Blocking CCL2/CCR2 signaling pathway can block the recruitment of monocytes and M2 polarization of tumour-associated macrophages (TAMs) in liver cancer [74]. The study showed that after injecting CCR2 antagonist into the abdominal cavity of mice with in-situ liver cancer model, the number of TAMs in liver cancer tumour was significantly reduced, while CD8+ T cells were significantly increased, which significantly inhibited tumour growth [75, 76]. Lentiviral knockdown of CCL2 or the CCR2 inhibitor CCX872 significantly attenuated ETV4-induced invasion of TAMs and MDSCs and metastasis of liver cancer. Previous studies have demonstrated that the CCL2/CCR2 axis promotes HCC invasion. Our study suggested that CCR2 was a risk factor for HCC. More RCT and functional research are needed to confirm these findings.

Naive CD4+ T cell Absolute Count typically represents a distinct subpopulation of immune cells characterised by the presence of CD4 co-receptors. CD4 is a receptor mainly located on the surface of helper T cells, playing a crucial role in the interaction with antigen-presenting cells (APC). Evaluating CD4+ cells can be critical in both scientific research and clinical settings. After receiving the activation signal of APC, naive CD4+T cells can differentiate into helper T cells with different functions according to different cytokines [77]. CD4+ T cells are pivotal in managing immune responses, as they secrete various cytokines upon activation and differentiation. There are several subtypes of CD4+ T

helper cells, such as T helper 1 (Th1), T helper 2 (Th2), T helper 17 (Th17), T helper 9 (Th9) and regulatory T (Treg) cells, each assuming distinct immunological roles after evolving from their naïve T cell state. The activation and development of different CD4+ T cell subsets depend on specific cytokines and key transcription factors [78]. Th1 is produced by IL-12 (and IFN-γ, IL-2) stimulation and differentiation, and secretes IFN-γ, IL-2, TNF-β and other cytokines, whose function is to stimulate cellular immunity, including macrophages, monocytes and CD8+T cells [79]. Th2 is mainly activated by IL-4, IL-2 and other cytokines, and secretes IL-4, IL-5, IL-9, IL-10, IL-13, IL-25 and other cytokines. The main transcription factors are STAT6 and GATA-3. The function is to stimulate B cells, eosinophils and mast cells to produce humoral immunity [80]. Th17 is differentiated by TGF-β, IL-6 and other cytokines, the main transcription factor is RORγt (also a TH17-specific molecule), secretes IL-17, IL-21, IL-22, GM-CSF and other cytokines, mainly activates and recruit neutrophils, responsible for clearing extracellular bacterial and fungal infections [81]. Th9 is also differentiated by TGF-β and IL-4 stimulation, expresses transcription factor PU.1, and mainly secretes cytokines such as IL-9 [82]. The function of regulatory T cells (Treg) is to inhibit immune activation, mainly induced by TGF-β. The surface of Treg and normal activated T helper cells express CD4 and CD25, but the difference is that Treg has its specific transcription factor FOXP3. The main function of Treg cells is to inhibit inflammation and autoimmune response, but also to promote tumour growth, as well as to cause some viruses can not be completely cleared, resulting in chronic infection [83]. Previous research has demonstrated that CD4+ T cells can be found in the tumour microenvironments of lung cancer, melanoma, CRC, lymphomas, cervical cancer and ovarian cancer, but the role of CD4+ T cells in EC is relatively understudied [84–89]. CD8+T cells are a key part of anti-tumour immunity, however, not only CD8+T cells play a key role in anti-tumour immunity, the anti-cancer effect of CD4+T cells can not be ignored. It has been found that the proliferation of CD8+ CTLS in liver cancer depends on cell triplets formed by progenitor cytlike CD8+T cells with DC cells and CD4+ helper T cells. The abundance of progenitor CD8+T cells and DC cells in tumours was similar between patients who responded to immunotherapy and those who did not, but there was significant enrichment of CD4+ helper T cells in patients who were effective [90]. In addition, at the boundary between tumour and normal tissue, some stromal cells or myeloid cells recruited by CD4+T cells can bypass MHC-I molecules and present tumour antigens to CD4+T cells via MHC-II molecules. CD4+T cells then further activate mononuclear macrophages, inducing inflammatory storms in the tumour and killing the tumour [91]. A previous study reported that naive CD4 + T cells were recruited and transformed into regulatory T cells (TREGs) by macrophage-derived CCL18 in breast cancer [92]. Recent studies have shown that naive CD4+T cells can convert to TreGs in specific states (cirrhosis and cancer), which may be related to macrophages, further confirming the link between naive CD4+T cell interactions in macrophages and the Treg population. However, our results suggest that the presence of naive CD4+ T cells is a protective factor for HCC, suggesting that the differentiation of naive CD4+ cells has a certain tendency in the immune environment of HCC tumours. More functional studies are needed to confirm these findings.

## Strength and limitations

In our MR study, we aimed to determine the causal links between various immune cells and HCC by analysing extensive GWAS data. This method provides an advantage over traditional observational studies by reducing confounders and avoiding the issue of reverse causation. Additionally, MR offers solutions to the limitations typically faced with representativeness and feasibility in RCTs.

Despite the strengths of our approach, there are several limitations to this study. First, reliance on publicly available GWAS datasets limited our ability to account for other potentially influential factors on HCC, such as sex, age and body mass index [93]. Second, the generalizability of our results is confined to Japanese populations, as they were the participants of the original GWAS. Extending the research to include diverse ethnic groups is necessary for a broader applicability of our findings [94].

Thirdly, despite performing multiple sensitivity analyses to ensure the robustness of our results, the possibility of horizontal pleiotropy was not entirely eliminated. Finally, we chose a broader threshold for significance to capture a more comprehensive picture of the relationship between immune cell profiles and HCC. While this approach may increase the risk of false positives, it simultaneously allows for a more extensive assessment of the potential associations.

## Conclusion

In summary, our MR analysis of the role of immune cells and HCC cancers provides a framework for characterising circulating immune status and suggests a dynamic immune cell environment in the setting of HCC. Our results also highlight multiple aspects of the immunosuppressive states found in HCC, all of which may serve as promising potential new therapeutic targets. Additional research is needed to confirm these findings and investigate the underlying mechanisms that affect the immune response to HCC.

## Conflicts of interest

The authors declare that they have no competing interests.

## Consent for publication

All authors of the article have given their consent for its publication in the journal.

## Patients consent for publication

Not applicable because we used public database to analyse this study.

## Ethics approval

Ethics approval is not applicable.

## Author contributions

Pengkhun Nov, Yangfeng Zhang: collected data, analysed, interpreted results and wrote the article; Kunpeng Du and Jiqiang Li: study design, revise and guide the study. The authors have read and approved the final manuscripts.

## Data availability

The data for this article is accessible through the GWAS database (https://www.ebi.ac.uk/gwas/).

## Supplementary tables

**Supplementary Table 1.** The pleiotropy of causal relationship between immune cells and HCC. The *p*-values for the MR-Egger intercept were above 0.05, suggesting that no significant pleiotropy effects were found.

exposure	egger_intercept	se	pval
CD64 on CD14- CD16+ monocyte	-0.001406728	0.100529992	0.989140758
CD3- lymphocyte Absolute Count	-0.126282238	0.063750074	0.088065458
CD3- lymphocyte %leukocyte	0.017189192	0.037076224	0.656992619
CD3- lymphocyte %lymphocyte	0.181699339	0.247018025	0.538558321
HLA DR on CD14+ monocyte	0.070029538	0.037184462	0.096414691
HLA DR on CD14+ CD16- monocyte	0.091156814	0.039802132	0.055787005
CD19 on Plasma Blast-Plasma Cell	0.031805562	0.027199477	0.272300917
CCR2 on monocyte	0.048454703	0.036669639	0.243606676
CCR2 on CD62L+ plasmacytoid Dendritic Cell	-0.022397981	0.027550695	0.437210135
Naive CD4+ T cell Absolute Count	-0.023350152	0.057010334	0.68997851
Naive CD4+ T cell %CD4+ T cell	-0.019718764	0.030465183	0.525210226
Naive CD4+ T cell %T cell	-0.027193901	0.034661104	0.444170131
Activated & resting CD4 regulatory T cell Absolute Count	-0.043274132	0.027329092	0.151978338
CD4 on Terminally Differentiated CD4+ T cell	0.021240508	0.016142373	0.207989823
Terminally Differentiated CD4+ T cell %CD4+ T cell	-0.043984742	0.03367558	0.211182962
HVEM on Terminally Differentiated CD4+ T cell	0.024939804	0.046860084	0.602919276
CD24 on IgD+ CD38- B cell	-0.021258024	0.03243389	0.526983227
IgD- CD27- B cell Absolute Count	-0.0038967	0.021485293	0.859379046
CD28+ CD45RA- CD8dim T cell %T cell	-0.003017453	0.013491548	0.827125174
HLA DR on CD33+ HLA DR+ CD14dim	0.03048143	0.03334665	0.382187037
Terminally Differentiated CD4+ T cell %T cell	-0.055469352	0.030457634	0.090011134
Granulocyte Absolute Count	-0.019564796	0.031213863	0.543580921
CD62L- monocyte %monocyte	-0.008903579	0.026732962	0.744027036
SSC-A on HLA DR+ CD4+ T cell	0.149822694	0.087428853	0.120740389
CD27 on CD20- CD38- B cell	0.008416388	0.022122167	0.712434893
CD40 on monocytes	-0.01222703	0.024321362	0.633071219
CD24 on unswitched memory B cell	-0.060571731	0.028463173	0.07741507
CD27 on T cell	0.052018227	0.044244227	0.278145805
Naive CD4-CD8- T cell %T cell	-0.003708493	0.051052007	0.943523826
CD28+ CD45RA+ CD8dim T cell Absolute Count	-0.019862514	0.012393588	0.134993655
Memory B cell Absolute Count	-0.0371556	0.120134926	0.769580142
CD8 on CD28+ CD45RA+ CD8+ T cell	-0.005871607	0.02746264	0.833782801
Unswitched memory B cell Absolute Count	0.020048673	0.027576217	0.494586037
CD28+ CD45RA+ CD8dim T cell %T cell	-0.010282828	0.011985507	0.403595583
SSC-A on granulocyte	-0.013777613	0.0182659	0.463180026
CD25++ CD4+ T cell Absolute Count	-0.001971578	0.018161947	0.917094477

**Supplementary Table 2.** The heterogeneity of causal relationship between immune cells and HCC. The *p*-values for the Cochran’s Q yielded were above 0.05, suggesting that no significant heterogeneity effects were found.

exposure	method	Q	Q_df	Q_pval
CD64 on CD14- CD16+ monocyte	MR Egger	3.687765179	9	0.93073615
CD64 on CD14- CD16+ monocyte	Inverse variance weighted	3.687960986	10	0.960327757
CD3- lymphocyte Absolute Count	MR Egger	4.96787559	7	0.663883674
CD3- lymphocyte Absolute Count	Inverse variance weighted	8.891822827	8	0.351503758
CD3- lymphocyte %leukocyte	MR Egger	3.776677187	7	0.805110565
CD3- lymphocyte %leukocyte	Inverse variance weighted	3.991618513	8	0.857878874
CD3- lymphocyte %lymphocyte	MR Egger	1.156555666	2	0.560863436
CD3- lymphocyte %lymphocyte	Inverse variance weighted	1.697620619	3	0.637462916
HLA DR on CD14+ monocyte	MR Egger	7.659553397	8	0.467412123
HLA DR on CD14+ monocyte	Inverse variance weighted	11.20637629	9	0.261827792
HLA DR on CD14+ CD16- monocyte	MR Egger	5.837478569	7	0.558849349
HLA DR on CD14+ CD16- monocyte	Inverse variance weighted	11.08272152	8	0.197055998
CD19 on Plasma Blast-Plasma Cell	MR Egger	9.97893666	9	0.352193106
CD19 on Plasma Blast-Plasma Cell	Inverse variance weighted	11.49503303	10	0.320271588
CCR2 on monocyte	MR Egger	2.262143198	5	0.811810648
CCR2 on monocyte	Inverse variance weighted	4.008200942	6	0.675566541
CCR2 on CD62L+ plasmacytoid Dendritic Cell	MR Egger	6.12132973	9	0.727716363
CCR2 on CD62L+ plasmacytoid Dendritic Cell	Inverse variance weighted	6.782255389	10	0.745829042
Naive CD4+ T cell Absolute Count	MR Egger	25.41676155	11	0.007918037
Naive CD4+ T cell Absolute Count	Inverse variance weighted	25.80437583	12	0.011438932
Naive CD4+ T cell %T cell	MR Egger	18.41726658	16	0.300035031
Naive CD4+ T cell %T cell	Inverse variance weighted	19.12580505	17	0.321359056
Naive CD4+ T cell %CD4+ T cell	MR Egger	22.14629623	19	0.277035935
Naive CD4+ T cell %CD4+ T cell	Inverse variance weighted	22.63461037	20	0.307089972
Activated & resting CD4 regulatory T cell Absolute Count	MR Egger	6.696929498	8	0.569656459
Activated & resting CD4 regulatory T cell Absolute Count	Inverse variance weighted	9.204229566	9	0.418639741
CD4 on Terminally Differentiated CD4+ T cell	MR Egger	5.960899108	15	0.980397076
CD4 on Terminally Differentiated CD4+ T cell	Inverse variance weighted	7.692289805	16	0.957503437
Terminally Differentiated CD4+ T cell %T cell	MR Egger	13.53856074	14	0.484622857
Terminally Differentiated CD4+ T cell %T cell	Inverse variance weighted	16.85531915	15	0.327586789
HVEM on Terminally Differentiated CD4+ T cell	MR Egger	19.22659726	14	0.156470278
HVEM on Terminally Differentiated CD4+ T cell	Inverse variance weighted	19.61560152	15	0.187179708
CD24 on IgD+ CD38- B cell	MR Egger	9.015776689	10	0.530606968
CD24 on IgD+ CD38- B cell	Inverse variance weighted	9.445360332	11	0.580855102
IgD- CD27- B cell Absolute Count	MR Egger	1.47189381	11	0.999653171
IgD- CD27- B cell Absolute Count	Inverse variance weighted	1.504787408	12	0.999867194
CD28- CD25++ CD8+ T cell %CD8+ T cell	MR Egger	10.59709851	12	0.563725767
CD28- CD25++ CD8+ T cell %CD8+ T cell	Inverse variance weighted	10.83015884	13	0.625043299
HLA DR on CD33+ HLA DR+ CD14dim	MR Egger	11.105421	10	0.349363964
HLA DR on CD33+ HLA DR+ CD14dim	Inverse variance weighted	12.0333212	11	0.36114379
Terminally Differentiated CD4+ T cell %T cell	MR Egger	13.53856074	14	0.484622857
Terminally Differentiated CD4+ T cell %T cell	Inverse variance weighted	16.85531915	15	0.327586789
Granulocyte Absolute Count	MR Egger	14.60441891	11	0.201332331
Granulocyte Absolute Count	Inverse variance weighted	15.12603048	12	0.234616464
CD62L- monocyte %monocyte	MR Egger	10.37142768	14	0.73455502
CD62L- monocyte %monocyte	Inverse variance weighted	10.48235413	15	0.788352575
SSC-A on HLA DR+ CD4+ T cell	MR Egger	13.77744769	9	0.130464993
SSC-A on HLA DR+ CD4+ T cell	Inverse variance weighted	18.27288458	10	0.050531265
CD27 on CD20- CD38- B cell	MR Egger	7.048877737	9	0.632030412
CD27 on CD20- CD38- B cell	Inverse variance weighted	7.193620262	10	0.707048315
CD40 on monocytes	MR Egger	6.214783352	6	0.399565234
CD40 on monocytes	Inverse variance weighted	6.476566014	7	0.485329804
CD24 on unswitched memory B cell	MR Egger	7.305681231	6	0.293500666
CD24 on unswitched memory B cell	Inverse variance weighted	12.81988523	7	0.076620367
CD27 on T cell	MR Egger	8.405753745	7	0.298177337
CD27 on T cell	Inverse variance weighted	10.06563301	8	0.260449535
CD28+ CD45RA+ CD8dim T cell Absolute Count	MR Egger	7.344045781	12	0.834065584
CD28+ CD45RA+ CD8dim T cell Absolute Count	Inverse variance weighted	9.912514663	13	0.701072233
Memory B cell Absolute Count	MR Egger	0.171439265	5	0.999391016
Memory B cell Absolute Count	Inverse variance weighted	0.267094776	6	0.999640746
CD8 on CD28+ CD45RA+ CD8+ T cell	MR Egger	23.8475084	14	0.047802575
CD8 on CD28+ CD45RA+ CD8+ T cell	Inverse variance weighted	23.92537377	15	0.066371653
Unswitched memory B cell Absolute Count	MR Egger	1.998706683	6	0.919817511
Unswitched memory B cell Absolute Count	Inverse variance weighted	2.527275899	7	0.925029196
CD28+ CD45RA+ CD8dim T cell %T cell	MR Egger	12.65980464	16	0.697449921
CD28+ CD45RA+ CD8dim T cell %T cell	Inverse variance weighted	13.3958631	17	0.709308668
SSC-A on granulocyte	MR Egger	14.35725691	14	0.423448429
SSC-A on granulocyte	Inverse variance weighted	14.94071448	15	0.455696057
CD25++ CD4+ T cell Absolute Count	MR Egger	1.818926634	6	0.93557584
CD25++ CD4+ T cell Absolute Count	Inverse variance weighted	1.83071091	7	0.968613205

**Supplementary Table 3.** The pleiotropy of reverse causal relationship between immune cells and HCC. The *p*-values for the MR-Egger intercept were above 0.05, suggesting that no significant pleiotropy effects were found.

outcome	egger_intercept	se	pval
Naive CD4+ T cell Absolute Count	0.021980732	0.032824029	0.513972176
Naive CD4+ T cell %CD4+ T cell	0.018394108	0.028382586	0.527421595
Naive CD4+ T cell %T cell	0.007525253	0.029710966	0.803731765
CCR2 on CD14- CD16-	0.061930855	0.030263883	0.059978773
CD45RA- CD4+ T cell %CD4+ T cell	-0.014506454	0.029040902	0.625172753
HLA DR on CD33+ HLA DR+ CD14dim	0.001622281	0.047778968	0.973393277
HLA DR on B cell	-0.015348952	0.036636262	0.681605644
CD4+ T cell %leukocyte	0.001110682	0.030012837	0.971002018
FSC-A on plasmacytoid Dendritic Cell	0.013757836	0.033536046	0.687838759
Central Memory CD4-CD8- T cell %CD4-CD8- T cell	-0.015869771	0.028596617	0.58768459
SSC-A on plasmacytoid Dendritic Cell	0.006124993	0.038986955	0.877406372
CD20 on switched memory B cell	-0.006080829	0.04017086	0.881840207
SSC-A on monocyte	0.029924114	0.034011845	0.393811717
CD20 on IgD- CD38dim B cell	0.011828603	0.039371508	0.768260009
SSC-A on Natural Killer T	-0.00213439	0.032990276	0.949329561
CD40 on monocytes	0.012201288	0.031006134	0.699869047
Basophil Absolute Count	-0.046839542	0.052100747	0.383845032
SSC-A on myeloid Dendritic Cell	0.010078304	0.043858588	0.821577719
FSC-A on monocyte	0.031360319	0.034982622	0.385165934
FSC-A on myeloid Dendritic Cell	0.001595846	0.041033819	0.969526441
FSC-A on granulocyte	0.046959633	0.034517357	0.195185614
CD4+ T cell %T cell	0.00211076	0.030274072	0.94540127

**Supplementary Table 4.** The heterogeneity of reverse causal relationship between immune cells and HCC. The p-values for the Cochran’s Q yielded were above 0.05, suggesting that no significant heterogeneity effects were found.

outcome	method	Q	Q_df	Q_pval
Naive CD4+ T cell Absolute Count	MR Egger	17.87125223	14	0.212707737
Naive CD4+ T cell Absolute Count	Inverse variance weighted	18.44368892	15	0.240058135
Naive CD4+ T cell %CD4+ T cell	MR Egger	10.66559146	14	0.712083653
Naive CD4+ T cell %CD4+ T cell	Inverse variance weighted	11.08559557	15	0.74650337
Naive CD4+ T cell %T cell	MR Egger	10.7320958	14	0.706942029
Naive CD4+ T cell %T cell	Inverse variance weighted	10.79624757	15	0.766899759
CCR2 on CD14- CD16-	MR Egger	7.734016898	14	0.902717679
CCR2 on CD14- CD16-	Inverse variance weighted	11.92161357	15	0.684952377
CD45RA- CD4+ T cell %CD4+ T cell	MR Egger	11.92489307	14	0.612334501
CD45RA- CD4+ T cell %CD4+ T cell	Inverse variance weighted	12.17441134	15	0.665782339
HLA DR on CD33+ HLA DR+ CD14dim	MR Egger	15.99838925	14	0.313472655
HLA DR on CD33+ HLA DR+ CD14dim	Inverse variance weighted	15.99970668	15	0.382071156
HLA DR on B cell	MR Egger	17.13541354	14	0.249033462
HLA DR on B cell	Inverse variance weighted	17.35024688	15	0.298361338
CD4+ T cell %leukocyte	MR Egger	13.71699689	14	0.471000893
CD4+ T cell %leukocyte	Inverse variance weighted	13.7183664	15	0.546987203
FSC-A on plasmacytoid Dendritic Cell	MR Egger	10.7739043	14	0.703699274
FSC-A on plasmacytoid Dendritic Cell	Inverse variance weighted	10.94220137	15	0.756680754
Central Memory CD4-CD8- T cell %CD4-CD8- T cell	MR Egger	13.7375871	14	0.469438524
Central Memory CD4-CD8- T cell %CD4-CD8- T cell	Inverse variance weighted	14.04555964	15	0.522076618
SSC-A on plasmacytoid Dendritic Cell	MR Egger	18.76332382	14	0.174191313
SSC-A on plasmacytoid Dendritic Cell	Inverse variance weighted	18.79640297	15	0.223108619
CD20 on switched memory B cell	MR Egger	24.05071393	14	0.045180261
CD20 on switched memory B cell	Inverse variance weighted	24.09007829	15	0.063579506
SSC-A on monocyte	MR Egger	10.57038977	14	0.719406515
SSC-A on monocyte	Inverse variance weighted	11.34446331	15	0.727815699
CD20 on IgD- CD38dim B cell	MR Egger	23.44612836	14	0.053386682
CD20 on IgD- CD38dim B cell	Inverse variance weighted	23.5972915	15	0.07225541
SSC-A on Natural Killer T	MR Egger	5.396359788	14	0.979496292
SSC-A on Natural Killer T	Inverse variance weighted	5.400545561	15	0.98815749
CD40 on monocytes	MR Egger	8.604712162	14	0.85550918
CD40 on monocytes	Inverse variance weighted	8.759563906	15	0.889749713
Basophil Absolute Count	MR Egger	22.90419111	14	0.061846683
Basophil Absolute Count	Inverse variance weighted	24.22647378	15	0.061346026
SSC-A on myeloid Dendritic Cell	MR Egger	22.80659239	14	0.06349051
SSC-A on myeloid Dendritic Cell	Inverse variance weighted	22.8926121	15	0.086446669
FSC-A on monocyte	MR Egger	14.90196416	14	0.384889006
FSC-A on monocyte	Inverse variance weighted	15.75736903	15	0.398357818
FSC-A on myeloid Dendritic Cell	MR Egger	20.45257691	14	0.116506166
FSC-A on myeloid Dendritic Cell	Inverse variance weighted	20.45478653	15	0.155171817
FSC-A on granulocyte	MR Egger	9.283330384	14	0.812483379
FSC-A on granulocyte	Inverse variance weighted	11.13419354	15	0.743024885
CD4+ T cell %T cell	MR Egger	13.51198569	14	0.486663804
CD4+ T cell %T cell	Inverse variance weighted	13.5168468	15	0.562442008

## References

[1] Llovet JM, Kelley RK, Villanueva A (2021). Hepatocellular carcinoma. Nat Rev Dis Primers.

[2] Huang DQ, El-Serag HB, Loomba R (2021). Global epidemiology of NAFLD-related HCC: trends, predictions, risk factors and prevention. Nat Rev Gastroenterol Hepatol.

[3] International Agency for Research on Cancer Estimated number of new cases in 2020, World, both sexes, all ages (excl. NMSC). Cancer Today.

[4] Siegel RL, Miller KD, Wagle NS (2023). Cancer statistics, 2023. CA Cancer J Clin.

[5] Llovet JM, Ricci S, Mazzaferro V (2008). SHARP Investigators Study Group Sorafenib in advanced hepatocellular carcinoma. N Engl J Med.

[6] Ray K (2017). Liver cancer: nivolumab: checkmate for hepatocellular carcinoma?. Nat Rev Gastroenterol Hepatol.

[7] El-Khoueiry AB, Sangro B, Yau T (2017). Nivolumab in patients with advanced hepatocellular carcinoma (CheckMate 040): an open-label, non-comparative, phase 1/2 dose escalation and expansion trial. Lancet.

[8] Gnjatic S, Bronte V, Brunet LR (2017). Identifying baseline immune-related biomarkers to predict clinical outcome of immunotherapy. J Immunother Cancer.

[9] Yang K, Li J, Zhao L (2022). Estimating the number of Chinese cancer patients eligible for and benefit from immune checkpoint inhibitors. Front Med.

[10] Taiji R, Cortes AC, Zaske AM (2023). Liver cancer vascularity driven by extracellular matrix stiffness: implications for imaging research. Invest Radiol.

[11] Hassan EA, Ahmed EH, Nafee AM (2019). Regulatory T cells, IL10 and IL6 in HCV related hepatocellular carcinoma after transarterial chemoembolization (TACE). Egypt J Immunol.

[12] Pingault JB, O’Reilly PF, Schoeler T (2018). Using genetic data to strengthen causal inference in observational research. Nat Rev Genet.

[13] Davies NM, Holmes MV, Davey SG (2018). Reading Mendelian randomisation studies: a guide, glossary, and checklist for clinicians. BMJ.

[14] Smith GD, Ebrahim S (2003). ‘Mendelian randomization’: can genetic epidemiology contribute to understanding environmental determinants of disease?. Int J Epidemiol.

[15] Emdin CA, Khera AV, Kathiresan S (2017). Mendelian randomization. JAMA.

[16] Orrù V, Steri M, Sidore C (2020). Complex genetic signatures in immune cells underlie autoimmunity and inform therapy. Nat Genet.

[17] Cai J, Li X, Wu S (2022). Assessing the causal association between human blood metabolites and the risk of epilepsy. J Transl Med.

[18] Zeng P, Wang T, Zheng J (2019). Causal association of type 2 diabetes with amyotrophic lateral sclerosis: new evidence from Mendelian randomization using GWAS summary statistics. BMC Med.

[19] Hartwig FP, Davey Smith G, Bowden J (2017). Robust inference in summary data Mendelian randomization via the zero modal pleiotropy assumption. Int J Epidemiol.

[20] Bowden J, Davey Smith G, Haycock PC (2016). Consistent estimation in Mendelian randomization with some invalid instruments using a weighted median estimator. Genet Epidemiol.

[21] Allen RJ, Porte J, Braybrooke R (2017). Genetic variants associated with susceptibility to idiopathic pulmonary fibrosis in people of European ancestry: a genome-wide association study. Lancet Respir Med.

[22] Burgess S, Bowden J, Fall T (2017). Sensitivity analyses for robust causal inference from Mendelian randomization analyses with multiple genetic variants. Epidemiology.

[23] Verbanck M, Chen CY, Neale B (2018). Detection of widespread horizontal pleiotropy in causal relationships inferred from Mendelian randomization between complex traits and diseases. Nat Genet.

[24] Hemani G, Tilling K, Davey SG (2017). Orienting the causal relationship between imprecisely measured traits using GWAS summary data. PLoS Genet.

[25] Hemani G, Zheng J, Elsworth B (2018). The MR-base platform supports systematic causal inference across the human phenome. Elife.

[26] Yavorska OO, Burgess S (2017). MendelianRandomization: an R package for performing Mendelian randomization analyses using summarized data. Int J Epidemiol.

[27] Nimmerjahn F, Ravetch JV (2008). Fcγ receptors as regulators of immune responses. Nat Rev Immunol.

[28] Amigorena S, Bonnerot C (1999). Fc receptor signalling and trafficking: a connection for antigen processing. Immunol Rev.

[29] García-García E, Rosales C (2002). Signal transduction during Fc receptor-mediated phagocytosis. J Leukoc Biol.

[30] Shi C, Pamer EG (2011). Monocyte recruitment during infection and inflammation. Nat Rev Immunol.

[31] Wong KL, Yeap WH, Tai JJ The three human monocyte subsets: implications for health and disease. Immunol Res.

[32] Luo Q, Xiao P, Li X (2018). Overexpression of CD64 on CD14++CD16- and CD14++CD16+ monocytes of rheumatoid arthritis patients correlates with disease activity. Exp Ther Med.

[33] Feng H, Yin J, Han YP (2015). Lymphocyte CD64 increased in patients with chronic HBV infection. Int J Clin Exp Med.

[34] Chiang CL, Ma Y, Hou YC (2023). Dual targeted extracellular vesicles regulate oncogenic genes in advanced pancreatic cancer. Nat Commun.

[35] Sun L, Beggs K, Borude P (2016). Bile acids promote diethylnitrosamine-induced hepatocellular carcinoma via increased inflammatory signaling. Am J Physiol Gastrointest Liver Physiol.

[36] Sangro B, Melero I, Wadhawan S (2020). Association of inflammatory biomarkers with clinical outcomes in nivolumab-treated patients with advanced hepatocellular carcinoma. J Hepatol.

[37] Galon J, Pagès F, Marincola FM (2012). The immune score as a new possible approach for the classification of cancer. J Transl Med.

[38] Galon J, Costes A, Sanchez-Cabo F (2006). Type, density, and location of immune cells within human colorectal tumors predict clinical outcome. Science.

[39] Eriksen AC, Sørensen FB, Lindebjerg J (2018). The prognostic value of tumor-infiltrating lymphocytes in stage II colon cancer. A nationwide population-based study. Transl Oncol.

[40] Kasurinen J, Hagström J, Kaprio T (2022). Tumor-associated CD3- and CD8-positive immune cells in colorectal cancer: the additional prognostic value of CD8+-to-CD3+ ratio remains debatable. Tumour Biol.

[41] Dos Santos NR, Ghysdael J, Tran Quang C (2019). The TCR/CD3 complex in leukemogenesis and as a therapeutic target in T-cell acute lymphoblastic leukemia. Adv Biol Regul.

[42] Xu X, Li H, Xu C (2020). Structural understanding of T cell receptor triggering. Cell Mol Immunol.

[43] Guy C, Mitrea DM, Chou PC (2022). LAG3 associates with TCR-CD3 complexes and suppresses signaling by driving co-receptor-Lck dissociation. Nat Immunol.

[44] Liu L, Cheng Y, Zhang F (2023). IL-2/GM-CSF enhances CXCR3 expression in CAR-T cells via the PI3K/AKT and ERK1/2 pathways. J Cancer Res Clin Oncol.

[45] Aboyoussef AM, Abdel-Sattar AR, Abdel-Bakky MS (2021). Enoxaparin prevents CXCL16/ADAM10-mediated cisplatin renal toxicity: role of the coagulation system and the transcriptional factor NF-κB. Life Sci.

[46] Wang D, Matsumoto R, You Y (2004). CD3/CD28 costimulation-induced NF-kappaB activation is mediated by recruitment of protein kinase C-theta, Bcl10, and IkappaB kinase beta to the immunological synapse through CARMA1. Mol Cell Biol.

[47] Magness ST, Jijon H, Van Houten Fisher N (2004). In vivo pattern of lipopolysaccharide and anti-CD3-induced NF-kappa B activation using a novel gene-targeted enhanced GFP reporter gene mouse. J Immunol.

[48] Hao S, Pan S, Hu J (2015). Aflatoxin B1 suppressed T-cell response to anti-pig-CD3 monoclonal antibody stimulation in primary porcine splenocytes: a role for the extracellular regulated protein kinase (ERK1/2) MAPK signaling pathway. J Agric Food Chem.

[49] Yu S, Zhang J, Yan Y (2019). A novel asymmetrical anti-HER2/CD3 bispecific antibody exhibits potent cytotoxicity for HER2-positive tumor cells. J Exp Clin Cancer Res.

[50] Bösmüller HC, Wagner P, Peper JK (2016). Combined immunoscore of CD103 and CD3 identifies long-term survivors in high-grade serous ovarian cancer. Int J Gynecol Cancer.

[51] Wu Z, Zhang Z, Lei Z (2019). CD14: biology and role in the pathogenesis of disease. Cytokine Growth Factor Rev.

[52] Lin Y, Dong J, Yu W (2023). CD14, a novel surface marker of esophageal cancer stem cells. Oncol Rep.

[53] Scheuermann RH, Racila E (1995). CD19 antigen in leukemia and lymphoma diagnosis and immunotherapy. Leuk Lymphoma.

[54] Liu HZ, Deng W, Li JL (2016). Peripheral blood lymphocyte subset levels differ in patients with hepatocellular carcinoma. Oncotarget.

[55] Pan J, Tang K, Luo Y (2023). Sequential CD19 and CD22 chimeric antigen receptor T-cell therapy for childhood refractory or relapsed B-cell acute lymphocytic leukaemia: a single-arm, phase 2 study. Lancet Oncol.

[56] Abramson JS (2020). Anti-CD19 CAR T-cell therapy for B-cell non-Hodgkin lymphoma. Transfus Med Rev.

[57] Leung WK, Ayanambakkam A, Heslop HE (2022). Beyond CD19 CAR-T cells in lymphoma. Curr Opin Immunol.

[58] Hu X, Manner K, DeJesus R (2023). Hypoimmune anti-CD19 chimeric antigen receptor T cells provide lasting tumor control in fully immunocompetent allogeneic humanized mice. Nat Commun.

[59] Jin X, Xu Q, Pu C (2021). Therapeutic efficacy of anti-CD19 CAR-T cells in a mouse model of systemic lupus erythematosus. Cell Mol Immunol.

[60] Denlinger N, Bond D, Jaglowski S (2022). CAR T-cell therapy for B-cell lymphoma. Curr Probl Cancer.

[61] Orlando EJ, Han X, Tribouley C (2018). Genetic mechanisms of target antigen loss in CAR19 therapy of acute lymphoblastic leukemia. Nat Med.

[62] Chen Y, Chang-Yong E, Gong ZW (2018). Chimeric antigen receptor-engineered T-cell therapy for liver cancer. Hepatobiliary Pancreat Dis Int.

[63] Bachelerie F, Ben-Baruch A, Burkhardt AM (2013). International Union of Basic and Clinical Pharmacology. [Corrected]. LXXXIX. Update on the extended family of chemokine receptors and introducing a new nomenclature for atypical chemokine receptors. Pharmacol Rev.

[64] Kurihara T, Warr G, Loy J (1997). Defects in macrophage recruitment and host defense in mice lacking the CCR2 chemokine receptor. J Exp Med.

[65] Marra F, Tacke F (2014). Roles for chemokines in liver disease. Gastroenterology.

[66] Seki E, Minicis S, Inokuchi S (2009). CCR2 promotes hepatic fibrosis in mice. Hepatology.

[67] Karlmark KR, Weiskirchen R, Zimmermann HW (2009). Hepatic recruitment of the inflammatory Gr1+ monocyte subset upon liver injury promotes hepatic fibrosis. Hepatology.

[68] Cochran BH, Reffel AC, Stiles CD (1983). Molecular cloning of gene sequences regulated by platelet-derived growth factor. Cell.

[69] Matsushima K, Larsen CG, DuBois GC (1989). Purification and characterization of a novel monocyte chemotactic and activating factor produced by a human myelomonocytic cell line. J Exp Med.

[70] Van Damme J, Proost P, Lenaerts JP (1992). Structural and functional identification of two human, tumor-derived monocyte chemotactic proteins (MCP-2 and MCP-3) belonging to the chemokine family. J Exp Med.

[71] Jia GQ, Gonzalo JA, Lloyd C (1996). Distinct expression and function of the novel mouse chemokine monocyte chemotactic protein-5 in lung allergic inflammation. J Exp Med.

[72] Garcia-Zepeda EA, Combadiere C, Rothenberg ME (1996). Human monocyte chemoattractant protein (MCP)-4 is a novel CC chemokine with activities on monocytes, eosinophils, and basophils induced in allergic and nonallergic inflammation that signals through the CC chemokine receptors (CCR)-2 and -3. J Immunol.

[73] Cao S, Liu M, Sehrawat TS (2021). Regulation and functional roles of chemokines in liver diseases. Nat Rev Gastroenterol Hepatol.

[74] Iwamoto H, Izumi K, Mizokami A (2020). Is the C-C motif ligand 2-C-C chemokine receptor 2 axis a promising target for cancer therapy and diagnosis?. Int J Mol Sci.

[75] Li X, Yao W, Yuan Y (2017). Targeting of tumour-infiltrating macrophages via CCL2/CCR2 signalling as a therapeutic strategy against hepatocellular carcinoma. Gut.

[76] Yao W, Ba Q, Li X (2017). A natural CCR2 antagonist relieves tumor-associated macrophage-mediated immunosuppression to produce a therapeutic effect for liver cancer. EBioMedicine.

[77] Shi G, Lovaas JD, Tan C (2012). Cell-cell interaction with APC, not IL-23, is required for naive CD4 cells to acquire pathogenicity during Th17 lineage commitment. J Immunol.

[78] Ashfaq H, Soliman H, Saleh M (2019). CD4: a vital player in the teleost fish immune system. Vet Res.

[79] Luckheeram RV, Zhou R, Verma AD (2012). CD4⁺T cells: differentiation and functions. Clin Dev Immunol.

[80] Yang W, Chen X, Hu H (2020). CD4+ T-cell differentiation in vitro. Methods Mol Biol.

[81] Soongsathitanon J, Homjan T, Pongcharoen S (2024). Characteristic features of in vitro differentiation of human naïve CD4+ T cells to induced regulatory T cells (iTreg) and T helper (Th) 17 cells: sharing of lineage-specific markers. Heliyon.

[82] Read KA, Powell MD, Sreekumar BK (2019). In vitro differentiation of effector CD4+ T helper cell subsets. Methods Mol Biol.

[83] Almeida-Santos J, Bergman ML, Demengeot J (2023). Differentiation of peripheral Treg. Methods Mol Biol.

[84] Kong CY, Sigel K, Criss SD (2018). Benefits and harms of lung cancer screening in HIV-infected individuals with CD4+ cell count at least 500 cells/μl. AIDS.

[85] Oliveira G, Stromhaug K, Cieri N (2022). Landscape of helper and regulatory antitumour CD4+ T cells in melanoma. Nature.

[86] Toor SM, Murshed K, Al-Dhaheri M (2019). Immune checkpoints in circulating and tumor-infiltrating CD4+ T cell subsets in colorectal cancer patients. Front Immunol.

[87] Niakosari F, Sur M (2007). Agranular CD4+/CD56+ hematodermic neoplasm: a distinct entity described in the recent World Health Organization-European Organization for Research and Treatment of Cancer classification for cutaneous lymphomas. Arch Pathol Lab Med.

[88] Li R, Liu Y, Yin R (2021). The dynamic alternation of local and systemic tumor immune microenvironment during concurrent chemoradiotherapy of cervical cancer: a prospective clinical trial. Int J Radiat Oncol Biol Phys.

[89] Ukita M, Hamanishi J, Yoshitomi H (2022). CXCL13-producing CD4+ T cells accumulate in the early phase of tertiary lymphoid structures in ovarian cancer. JCI Insight.

[90] Magen A, Hamon P, Fiaschi N (2023). Intratumoral dendritic cell-CD4+ T helper cell niches enable CD8+ T cell differentiation following PD-1 blockade in hepatocellular carcinoma. Nat Med.

[91] Kruse B, Buzzai AC, Shridhar N (2023). CD4+ T cell-induced inflammatory cell death controls immune-evasive tumours. Nature.

[92] Su S, Liao J, Liu J (2017). Blocking the recruitment of naive CD4+ T cells reverses immunosuppression in breast cancer. Cell Res.

[93] Tian D, Zhou Y, Chen Y (2023). Genetically predicted ankylosing spondylitis is causally associated with psoriasis. Front Immunol.

[94] Zhao N, Guo P, Tang M (2023). Evidence for a causal relationship between psoriasis and cutaneous melanoma: a bidirectional two-sample Mendelian randomized study. Front Immunol.

